# Word selection affects perceptions of synthetic biology

**DOI:** 10.1186/1754-1611-5-9

**Published:** 2011-07-21

**Authors:** Brianna Pearson, Sam Snell, Kyri Bye-Nagel, Scott Tonidandel, Laurie J Heyer, A Malcolm Campbell

**Affiliations:** 1 Department of Biology, Davidson College, Davidson, NC 28035, USA; 2 Department of Psychology, Davidson College, Davidson, NC 28035, USA; 3 Department of Sociology, Davidson College, Davidson, NC 28035, USA; 4 Department of Mathematics, Davidson College, Davidson, NC 28035, USA

**Keywords:** synthetic biology, ethics, education, religiosity, framing effects, public perception

## Abstract

Members of the synthetic biology community have discussed the significance of word selection when describing synthetic biology to the general public. In particular, many leaders proposed the word "create" was laden with negative connotations. We found that word choice and framing does affect public perception of synthetic biology. In a controlled experiment, participants perceived synthetic biology more negatively when "create" was used to describe the field compared to "construct" (*p *= 0.008). Contrary to popular opinion among synthetic biologists, however, low religiosity individuals were more influenced negatively by the framing manipulation than high religiosity people. Our results suggest that synthetic biologists directly influence public perception of their field through avoidance of the word "create".

## 

In response to public concern about the production of the first "synthetic cell" in 2010, President Obama instructed the U.S. Bioethics Commission to scrutinize the ethics of synthetic biology [1]. While the commission reported synthetic biology research could continue, they recommended progressing with extreme caution. During this same time, some religious leaders claimed synthetic biology was dangerously close to "pretending to be God". The Italian bishops' legal affairs committee chairman, Bishop Domenico Mogavero, said, "Pretending to be God and parroting his power of creation is an enormous risk that can plunge men into a barbarity." [2]

Like other technologies, synthetic biology and society profoundly influence each other. The actions of scientists determine the level of public support, and scientists, corporations, and society at large must collaborate and address obstacles at the heart of communication, learning from previous controversial technologies. How does word choice affect public perception of synthetic biology? Previous literature has described the power of word choice as "framing effects" [3]. Nisbet and Scheufule [4] described frames as being used by three constituencies: 1) "audiences to make sense of and discuss an issue; 2) journalists to craft interesting and appealing news reports; and 3) policymakers to define policy options and reach decisions." Depending on political interests, religion, and gender, etc., people allow frames "to hold particular sway... because frames reduce confusing issues that are remote from most people's direct experiences into manageable packages of understandable information." [5] When synthetic biologists use the word "creation" to describe their products, some people might find the work to be offensive or blasphemous because of the religious power that term evokes. An article from the British *Daily Mail *described the production of a "synthetic cell" as the "second genesis" and quoted synthetic biologist Craig Venter as having changed his own perception of life since he essentially, "allowed a new creature to enter the world." A poll associated with the *Daily Mail *article asked, "Should scientists be allowed to create synthetic life?" Sixty percent of the respondents opposed Venter's research when framed in this way [6]. Similarly, views of adult and embryonic stem cell research are found to be negatively correlated with church attendance (*p *< 0.01)[7].

To address the framing of synthetic biology, 100 participants from a variety of backgrounds were randomly presented with one of two different written descriptions of synthetic biology (see additional file 1). One description included the word "create" while the other used the word "construct". Using an independent samples t-test, we found that framing significantly affected public perception of synthetic biology (t (79) = 2.69, p = .008, d = .30; Figure 1) such that individuals presented with the "create" description held a more negative perception of synthetic biology than individuals presented with the "construct" description. This significant result is compelling given the otherwise high degree of similarity between the two descriptions of synthetic biology and the subtlety of the framing manipulation.

**Figure 1:**
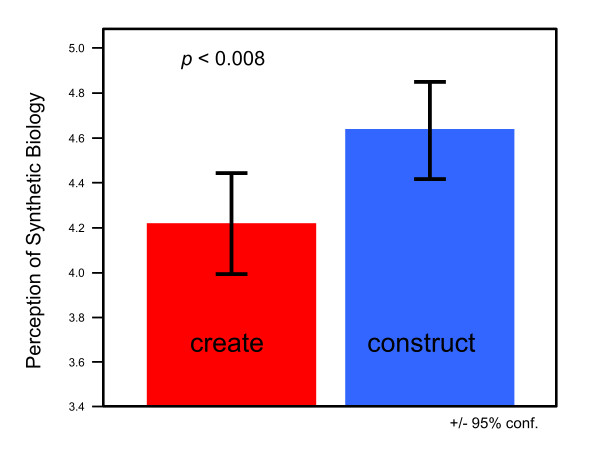
**Perception of synthetic biology based on whether "create" or "construct" manipulation was presented (based on scale of 1-6 with 6 indicating more positive perception of synthetic biology)**. Error bars represent 95% confidence interval (see additional file 1). N = 81.

We also examined whether the effect of framing on perceptions of synthetic biology differed as a function of participant religiosity [8-10]. To evaluate this, we used moderated multiple regression (MMR) where perceptions of synthetic biology were regressed on three predictors: the framing manipulation, religiosity, and the interaction between the two. As is customary in MMR, the continuous predictor was mean centered prior to the analysis. The interaction between religiosity and the framing manipulation is shown in Figure 2. Since religiosity is a continuous predictor, to generate the plot we used values of 1.5 standard deviations above and below the mean to indicate high vs. low religiosity. Those values along with the values associated with the different conditions of the framing manipulation were entered into the regression equation obtained above to generate the plot. Though the interaction was not statistically significant by conventional standards (*p *= 0.09), we were surprised to find that individuals low in religiosity were more influenced by the framing manipulation than people scoring higher on the religiosity scale. Perhaps people categorized with a high religiosity are less likely to be swayed by external factors such as the framing manipulated in our experiment. Our results are consistent with a study looking at the public's evaluation of stem cell research. Stewart *et al*. found that attending religious services was correlated with more positive evaluation of adult stem cell research [11]. Stewart *et al*. concluded, "Individuals' beliefs about the relationship between science and religion, rather than their religious attendance, are more important in making evaluations about the ethics and usefulness of embryonic stem cell research."

**Figure 2:**
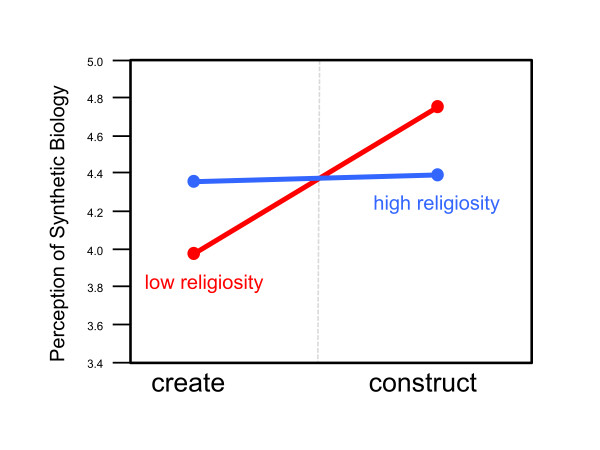
**Effect of religiosity on perceptions of synthetic biology as a function of whether "create" or "construct" manipulation was presented (based on a scale of 1-6 with 6 indicating more positive perception of synthetic biology; see additional file 1).**[Supplementary-material S1] N = 81.

Synthetic biology is a young discipline that could better influence its perception by society. To minimize negative perception, investigators might consider using the term "construct" rather than "create" when describing their work. We were surprised to learn that individuals scoring lower in religiosity were more likely to be influence by word choice than those with higher religiosity scores. Contrary to the perception of many synthetic biologists, low religiosity people are more easily swayed by the word "create" and thus investigators should avoid using "create" regardless of the audience.

## Competing interests

The authors declare that they have no competing interests.

## Authors' contributions

SS and ST designed the surveys, collected and analyzed the data. KBN conducted follow up interviews. BP wrote the manuscript. LJH and AMC conceived of the study, and participated in its design and coordination. All authors read and approved the final manuscript.

## Supplementary Material

Additional File 1 **Methods for data collection and analysis**. Data collection and analysis for the surveys conducted on individual reaction to create vs. construct framing. Click here for file
