# Supplementary material for: Word selection affects perceptions of synthetic biology
Source: J Biol Eng. 2011 Jul 21;5:9. doi: 10.1186/1754-1611-5-9 (PMC3148555; doi:10.1186/1754-1611-5-9)
Supplement: Additional File 1 — Methods for data collection and analysis. Data collection and analysis for the surveys conducted on individual reaction to create vs. construct framing. [file 1754-1611-5-9-S1.DOC]

**Methods for Data Collection and Analysis**

To measure framing effects and public perceptions, we conducted a survey of 100 individuals from greater Charlotte, North Carolina (USA) area. Forty-five participants received the “construct” manipulation. Sixty-eight percent of these participants were women, and the mean age of these participants was 49.22 years (SD = 18.35). Fifty-five participants received the “create” manipulation. Sixty-two percent of these participants were women, and the mean age of these participants was 46.84 years (SD = 17.49). Eighty-seven percent of participants in the “construct” manipulation and 80 percent of participants in the “create” manipulation reported having at least a Bachelors degree. Because some participants failed to respond to all of the items on the survey, the sample size associated with particular analyses varies.

The study utilized a 2 x 2 between-subjects design. The two independent variables were participant religiosity (high vs. low) and the randomly assigned manipulation (“construct” vs. “create”). Participants’ perception of synthetic biology was the dependent variable. Surveys were advertised and administered through two media: paper and electronic. With both media, participants self-selected into the study, and no incentives were offered for completion. Furthermore, participants were given no indication as to the manipulation group into which he or she had been randomly assigned, and the manipulated description of synthetic biology was presented before participants were asked to rate their perception of the discipline.

Paper surveys were distributed to 20 libraries and displayed at the circulation desk alongside a brief advertisement. Surveys were arranged in the display case such that the manipulation condition randomly varied from one survey to the next. Adjacent to the display case sat an opaque envelope in which participants could place their completed surveys. Library managers secured completed surveys at the end of each business day and hand-delivered all completed surveys to the research team upon completion of the study. Responses were entered into two identical databases during two independent sessions. A comparison between the two databases confirmed that responses were entered correctly.

Electronic surveys had a layout identical to that of the paper surveys but were administered via an online format. Electronic surveys were advertised on a local news website, from which participants could click on a hyperlink to access the survey. Participants were randomly assigned to one of the two manipulation groups and subsequently shown the corresponding survey. Responses were recorded and collected electronically.

Participants’ perception of synthetic biology was measured using a series of nine semantic differentials. Each of the nine semantic differentials asked the participant to choose the degree to which they thought synthetic biology coincided with a negatively charged word versus a positively charged word (*e.g.,* good vs. bad, safe vs. dangerous, ethical vs. unethical). Participants were provided six response options that allowed them to express their relative agreement with the connotation of each word as applied to synthetic biology. The internal consistency of these items was 0.83.

The manipulations used in this study were designed to frame synthetic biology from two contrary but equally valid perspectives. The descriptions used in each manipulation group were developed alongside subject matter experts in synthetic biology. Additionally, the descriptions were piloted on synthetic biology students as well as on members of the public who had no experience in synthetic biology. Each description contained the same core information in five or six sentences, but key phrases within each description were manipulated to describe synthetic biology as either “creating” modified living organisms or as “constructing” novel biological devices. The description of synthetic biology associated with the “construct” frame was:

*Synthetic biology uses molecular methods to construct DNA-based devices to perform novel functions. Synthetic biologists design, model, and construct biological parts, devices and systems by engineering and modifying existing biological systems. For instance, synthetic biologists can produce microbes that make useful chemicals. Synthetic biology gives scientists the tools to build biological devices from smaller biological units in much the same way as an engineer constructs a building from smaller construction materials. Synthetic biology has the potential to develop technologies that could lead the way in the 21st century.*

The description of synthetic biology used for the “create” manipulation was:

*Synthetic biology uses molecular methods to create modified living organisms that have never existed before. These DNA-based devices perform new functions not found in nature. Synthetic biologists redesign and modify existing, natural biological systems. For instance, synthetic biologists can create new versions of microbes that make chemicals not naturally found in microbes. In many ways, synthetic biology allows scientists to redesign biological processes and create organisms not found in nature. Synthetic biology has the potential to develop technologies that could lead the way in the 21st century.*

A multidimensional religiosity scale was developed in accordance with recommendations put forth by Wilkes, Burnett, and Howell [8], and similar measures have been adapted for fields as diverse as elderly depression and economic decision-making. [9,10] The religiosity scale contained four items measuring the following dimensions: religious service attendance, importance of religious values, confidence in religious values, and self-perceived religiosity. For example, one item on the scale was “Spiritual values are more important than material things.” The four items were measured on a seven-point response scale from 1 (strongly disagree) to 7 (strongly agree). The internal consistency of this measure was 0.85.
